# Socioeconomic position and prognosis in premenopausal breast cancer: a population-based cohort study in Denmark

**DOI:** 10.1186/s12916-021-02108-z

**Published:** 2021-09-30

**Authors:** Cathrine Fonnesbech Hjorth, Per Damkier, Bent Ejlertsen, Timothy Lash, Henrik Toft Sørensen, Deirdre Cronin-Fenton

**Affiliations:** 1 Department of Clinical Epidemiology, Department of Clinical Medicine, Aarhus University and Aarhus University Hospital, Olof Palmes Allé 43-45, 8200 Aarhus N, Denmark; 2 Department of Clinical Biochemistry and Pharmacology, J.B. Winsløvs vej 4, Odense University Hospital, 5000 Odense, Denmark; 3 Department of Clinical Research, Winsløwparken 19, University of Southern Denmark, 5000 Odense, Denmark; 4 Danish Breast Cancer Group, Blegdamsvej 9, 2100 Copenhagen, Denmark; 5 Department of Oncology, University of Copenhagen, Blegdamsvej 9, Rigshospitalet, 2100 Copenhagen, Denmark; 6 Department of Epidemiology, Rollins School of Public Health, Emory University, 1518 Clifton Rd, Atlanta, GA 30322 USA

**Keywords:** Socioeconomic position, Social inequality, Breast cancer, Survivorship, Taxanes, Docetaxel, Recurrence, Mortality, Survival analyses

## Abstract

**Background:**

To investigate how socioeconomic position (SEP) influences the effectiveness of cancer-directed treatment in premenopausal breast cancer patients in terms of breast cancer recurrence and mortality.

**Methods:**

We conducted a cohort study nested in the ProBeCaRe (Predictors of Breast Cancer Recurrence) cohort (*n* = 5959). We identified all premenopausal women aged 18–55 years diagnosed with non-metastatic breast cancer and prescribed docetaxel-based chemotherapy in Denmark during 2007–2011. Population-based administrative registries provided data on SEP: marital status (married including registered partnership or single including divorced or widowed), cohabitation (cohabiting or living alone), education (low, intermediate, or high), income (low, medium, or high), and employment status (employed, unemployed, or health-related absenteeism). For each SEP measure, we computed incidence rates, cumulative incidence proportions (CIPs), and used Poisson regression to compute incidence rate ratios (IRRs) and 95% confidence intervals (CIs) of recurrence and death. We stratified on estrogen receptor (ER) status/tamoxifen to evaluate interaction.

**Results:**

Our study cohort included 2616 women; 286 (CIP 13%) experienced recurrence and 223 (CIP 11%) died during follow-up (median 6.6 and 7.2 years, respectively). Single women had both increased 5-year risks of recurrence (IRR 1.45, 95% CI 1.11–1.89) and mortality (IRR 1.83, 95% CI 1.32–2.52). Furthermore, we observed increased 5-year mortality in women with low education (IRR 1.49, 95% CI 0.95–2.33), low income (IRR 1.37, 95% CI 0.83–2.28), unemployment (IRR 1.61, 95% CI 0.83–3.13), or health-related work absenteeism (IRR 1.80, 95% CI 1.14–2.82), but smaller or no increased risk of recurrence. These findings were especially evident among women with ER+ tumors prescribed tamoxifen. Overall analyses (follow-up max. 10 years) provided similar results.

**Conclusions:**

Low SEP in premenopausal women with non-metastatic breast cancer was associated with increased mortality, but not always recurrence. This suggests underdetection of recurrences in certain groups. Poor prognosis in women with low SEP, especially single women, may partly be explained by tamoxifen adherence.

**Supplementary Information:**

The online version contains supplementary material available at 10.1186/s12916-021-02108-z.

## Background

Advances in breast cancer screening and treatment have enlarged the pool of breast cancer survivors [1]. These women are at risk of breast cancer recurrence and premature death, but data on how socioeconomic position (SEP) influences this risk are scarce.

SEP has been associated with breast cancer recurrence, but most studies stem from settings of unequal healthcare access and non-uniform insurance coverage [2–6]. Furthermore, most studies focused on racial, ethnic, or insurance-related disparities [2–5]. Countries with tax-funded healthcare could be expected to offer equal treatment and follow-up after cancer irrespective of SEP. However, in Nordic countries, health inequality persists despite the build-up of the welfare states (also called the Nordic paradox) [7]. Studies set in Denmark, a country with tax-funded healthcare, report increased risks of breast cancer recurrence (and other cancer types) among patients with low education and in those living alone [8], and a higher 5-year mortality in women with lower education and income [9, 10], even after adjusting for tumor characteristics, treatment, and comorbidities [11, 12]. The same tendency has been observed in other European countries [12]. Thus, SEP-related disparities in breast cancer prognosis are evident even in populations with universal healthcare access.

The potential influence of SEP on breast cancer prognosis may differ according to patients’ characteristics. Scandinavian studies show increased mortality in women with breast cancer with low SEP aged below 50 years (assumed premenopausal) [13–15], whereas no such association was seen in their older counterparts [13, 14]. Moreover, stage-specific survival has improved less over time in Norwegian women with low SEP, compared to women with high SEP [15].

Breast tumors in premenopausal women often have poor prognostic characteristics—higher stage and grade and estrogen receptor (ER) negative [16, 17]. Accordingly, most premenopausal women are recommended taxane-based chemotherapy, followed by up to 10 years of tamoxifen therapy for those with ER+ tumors, whereas postmenopausal women to a greater extent can forego chemotherapy [18–20]. Yet, studies on breast cancer prognosis according to SEP did not distinguish pre- from postmenopausal breast cancer [8–11], lacked treatment-specific information [8, 15, 21, 22], failed to stratify by ER status [8–11, 14, 15, 21, 22], and included patients with metastatic breast cancer [11, 13, 14, 22, 23]. We therefore investigated the association between individual-level SEP and breast cancer recurrence and overall mortality in a contemporary cohort of Danish premenopausal women with non-metastatic breast cancer, treated with taxane-based chemotherapy. We evaluated interaction by ER status and associated tamoxifen therapy.

## Methods

### Setting and design

This nationwide population-based cohort study was conducted in Denmark, a country with unfettered access to tax-financed healthcare [24]. All Danish citizens and legal residents have a Civil Personal Registration number, which facilitates individual-level data linkage across all Danish administrative and health registries [24]. Since 1977, clinical guidelines for diagnosis, treatment, and follow-up in breast cancer patients in Denmark have been directed by the Danish Breast Cancer Group (DBCG), informed by international research and DBCG clinical trials [25]. In their clinical database, DBCG registers follow-up data for all patients with invasive breast cancer [26]. Completeness is achieved by means of data linkage to the Danish Pathology Registry recording first primary tumors [26], and electronic reporting from all Danish pathology departments, supported by a query reminder system [27]. DBCG’s follow-up data includes data on recurrences detected at follow-up exams that until 2015 took place semi-annually in the first 5 years and annually in the next 5 years for patients on active treatment [28]. Since 2015, patients have been offered structured follow-up at 12 and 18 months and then given a choice of physician-, nurse-, or patient-led follow-up for a total of 10 years. However, all breast cancer survivors are referred to a mammography screening program and are expedited through diagnostic work-up in the case of new symptoms [29].

### Study cohort

We nested our study cohort in the ProBeCaRe (Predictors of Breast Cancer Recurrence) cohort [17], which includes premenopausal women diagnosed with incident non-metastatic breast cancer in Denmark during 2002–2011, registered in the DBCG clinical database. We restricted the study cohort to women aged 18–55 years diagnosed during 2007–2011 and treated with adjuvant chemotherapy, as current guideline treatment including taxane-based chemotherapy was introduced on 1 January 2007.

### Data collection from Danish registries

We collected SEP information from administrative registries, marital status and cohabitation from the Danish Civil Registration System [24], income from the Danish Income Statistics Registry [30], highest completed education from the Population’s Education Registry [31] and social security payment data from the Danish Register for Evaluation of Marginalization to assess employment status [32]. From the DBCG clinical database, we obtained information on age, tumor characteristics, and treatments, and date of recurrence or other malignancies. Last, we retrieved information on emigration from Statistics Denmark, dates and cause of death from the Cause of Death Registry [33], and comorbidities from the Danish National Patient Registry [34].

### Analytic variables

Covariate assessment according to the underlying time scale is graphically illustrated in Additional file 1: Figure S1 [35].

### Socioeconomic position

We collected information on SEP at breast cancer diagnosis, or during the 2 years preceding diagnosis, to avoid reverse causation [36]. We categorized marital status at diagnosis as married, including women living in a registered partnership, or single, including never married, divorced, or widowed. We considered cohabitation status in the calendar year before the year of breast cancer diagnosis as cohabiting (living with a partner) or living alone. Income reflected the total household income minus total taxes accounting for the number of persons in the household. We defined income as the average income in the two preceding calendar years using sample quartiles (Q) categorized into low (< Q1), medium (Q1–Q2), and high (< Q3). We categorized education into low (~ 9–11 years of schooling), intermediate (~ 12–13 years of schooling), and high (~ 14–20 year of schooling) (Additional file 1: Figure S2). Last, we collected weekly employment data 3–12 months before breast cancer diagnosis and categorized it by the main presence of employment, unemployment, or health-related absenteeism (Additional file 1: Table S1). Please see Additional file 1: Figure S3 for a detailed description of the interrelated relationships between the SEP measures and their relationship with other covariates.

### Recurrence and death

We used the DBCG’s definition of breast cancer recurrence as locoregional or distant recurrence or contralateral breast cancer diagnosed up to 10 years after diagnosis [37]. We defined overall mortality as deaths from any cause, as deaths in these young women were likely related to breast cancer.

### Covariates

We included a comprehensive set of covariates: age, pathological stage I–III according to the TNM (tumor, node, metastasis) staging system [38], grade 1–3 in ductal and lobular tumors, received surgery type categorized as lumpectomy (including intended radiation therapy) or mastectomy, ER status, human epidermal growth factor 2 (HER2) status, and triple negative breast cancer if tumors were ER–, HER2–, and had negative or missing progesterone receptor status. All women with ER+ tumors were prescribed tamoxifen therapy, as we only included women on guideline treatment protocols [17]. We used the Charlson comorbidity index (CCI) [39] to summarize comorbidities diagnosed before breast cancer diagnosis (Additional file 1: Table S2).

### Statistical analyses

We described the study cohort calculating distributions and person years across all covariates, including missings. Follow-up time began 6 months after the date of diagnosis (date of surgery), equal to the time period during which most women in our cohort would likely have completed chemotherapy. We followed the women until recurrence/death, emigration, or 31 December 2016. When examining recurrence, we censored at other malignancy, last follow-up visit, or end of DBCG protocol (maximum 10 years). We calculated median follow-up time (incorporating the 6 months after surgery) including the interquartile range (IQR). To assess the incidences of recurrence and death, we calculated cumulative incidence proportions (CIPs) and presented these using the Nelson-Aalen estimator, treating death as a competing risk when examining recurrence. We calculated incidence rates (IRs) per 1000 person years by dividing the number of events by risk time and used Poisson regression models to compute crude and directed acyclic graph (DAG) [40] adjusted incidence rate ratios (IRR) (Additional file 1: Figure S3). We stratified by ER status to evaluate interaction. Post hoc, we evaluated potential interaction by stratifying models of marital status by cohabitation. All statistical analyses were conducted using SAS software, Version 9.4.

### Sensitivity analyses

To account for potential underreporting of recurrence [41], we changed our definition of recurrence to also include breast cancer-specific mortality (BCSM) (in those with no recurrence registered), expecting those to be underreported recurrences. We also examined BCSM separately to validate our mortality results. Last, we changed the time frame of employment status assessment to 1–3 months before breast cancer diagnosis.

## Results

The ProBeCaRe cohort included 5959 premenopausal women diagnosed during 2002–2011; 2979 were diagnosed after 1 January 2007. After exclusions, our study cohort included 2616 women (Additional file 1: Figure S4). Table 1 illustrates the characteristics of the study cohort. A total of 286 women (CIP 13%) were diagnosed with a recurrence over a median 6.6 years (IQR 5.4–7.9) of follow-up; 97% (*n* = 227) of these were diagnosed in the first 5 years (CIP 9%). Two hundred twenty-three women (CIP 11%) died during a median follow-up of 7.2 years (IQR 6.0–8.6), 66% (*n* = 148) within 5 years (CIP 6%) (Additional file 1: Figure S5).

**Table 1:** Descriptive patient characteristics of premenopausal women diagnosed with non-metastatic breast cancer during 2007-2011

	N	%	Person years
Overall	2616	100	15605
Marital status ^a^			
Married	1709	65.3	10395
Single	907	34.7	5210
Cohabitation			
Living with partner	2006	76.7	12073
Living alone	588	22.5	3394
Missing	22	0.8	139
Household income ^b^			
Low	651	24.9	3808
Medium	1304	49.8	7951
High	652	24.9	3789
Missing	9	0.3	57
Education level ^c^			
Low	436	16.7	2587
Intermediate	1107	42.3	6671
High	1048	40.1	6198
Missing	25	1.0	149
Employment status ^d^			
Employed	2212	84.6	13343
Unemployed	116	4.4	654
Health-related absenteeism	280	10.7	1561
Not resident in Denmark	8	0.3	48
Age at diagnosis ^e^			
<35 years	190	7.3	1064
35-44 years	990	37.8	5954
45-55 years	1436	54.9	8587
CCI score			
0	2279	87.1	13702
1-2	252	9.6	1457
3 or more	85	3.2	446
Surgery type			
Mastectomy	1019	39.0	5923
Lumpectomy^f^	≤1600		
Missing	≤5		
ER status			
Negative	573	21.9	3093
Positive	2043	78.1	12512
HER2 status			
Negative	1971	75.3	11670
Positive	479	18.3	2829
Unknown	166	6.3	1107
TNBC			
No	2218	84.8	13503
Yes	296	11.3	1602
Unknown	102	3.9	501
Positive lymph nodes			
None	1045	39.5	6374
1-2	1125	43.0	6840
3 or more	434	16.6	2340
Missing	12	0.5	51
Tumor size (mm)			
≤ 20	≤1428		
21-50	1096	41.9	6462
51 or above	88	3.4	474
Missing	≤5		
TNM stage ^g^			
Stage I	671	25.6	4079
Stage II	1463	55.9	8932
Stage III	462	17.7	2494
Missing	20	0.8	100
Pathological grade ^h^			
Grade 1	393	15.0	2498
Grade 2	1091	41.7	6716
Grade 3	860	32.9	4806
Not graded	240	9.2	1404
Missing	32	1.2	181

^a^ Married included women living in a registered partnership; single included never married, divorced, or widowed. ^b^ Based on sample quartiles categorized into low (<Q1), medium (Q1-Q2), and high (Q3<). ^c^ Low: primary school (~9-11 years of schooling); intermediate: upper secondary school (~12-13 years of schooling); high: tertiary education (~14-20 year of schooling). ^d^ Employed included women receiving no benefits and women on maternity leave; unemployment included women receiving non-health related unemployment benefits; health-related absenteeism included women receiving a health-related social benefit. ^e^ Median age was 45 years (IQR: 41–49).^f^ Including intention to treat radiotherapy. If lumpectomy was followed by mastectomy, the date of lumpectomy was used ^g^ Derived from tumor size and lymph node status. ^h^ Ductal and lobular tumors. Other were not graded. In accordance with Danish law, cell sizes with fewer than five observations and cells permitting back calculation are reported in aggregate

Abbreviations: *CCI* Charlson Comorbidity Index, *ER* estrogen receptor, *HER2* human epidermal growth factor 2, *TNBC* triple negative breast cancer, *TNM* tumor, node, metastasis

### Socioeconomic position

**Fig. 1:**
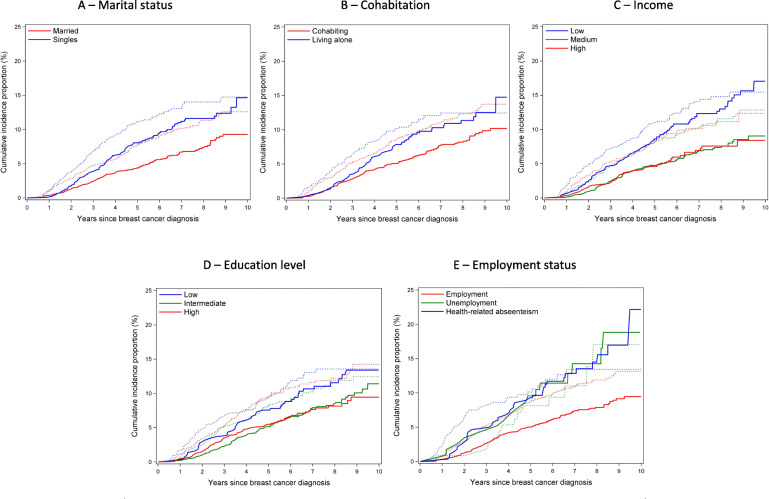
Cumulative incidence of recurrence and mortality by socioeconomic position among premenopausal women with non-metastatic breast cancer. Solid lines illustrate mortality and dashed lines illustrate recurrence with color-assigned strata. Follow-up started 6 months after diagnosis, illustrated by the flat curves in this period

**Fig. 2:**
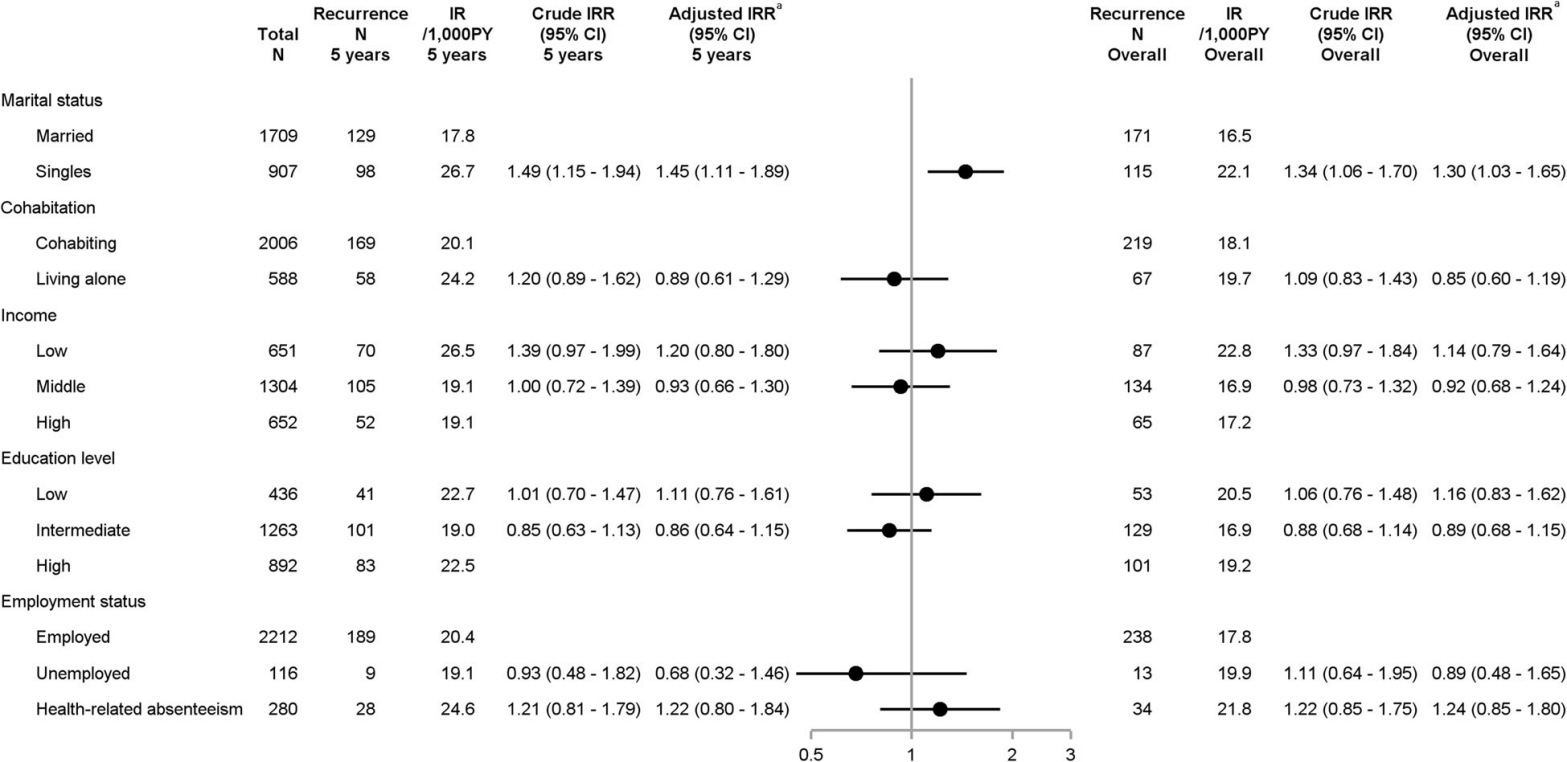
Incidence rates and incidence rate ratios of breast cancer recurrence by socioeconomic position. Plots illustrate 5-year adjusted IRRs and error bars 95% CIs. Superscript lowercase letter “a” indicates the following: marital status was adjusted for age; cohabitation for age and marital status; income for age, comorbidities, marital status, cohabitation, education, and employment; education for age; employment for age, comorbidities, and education. Abbreviations: CI, confidence interval; IR, incidence rate; IRR, incidence rate ratio; N, numbers; PY, person years

**Fig. 3:**
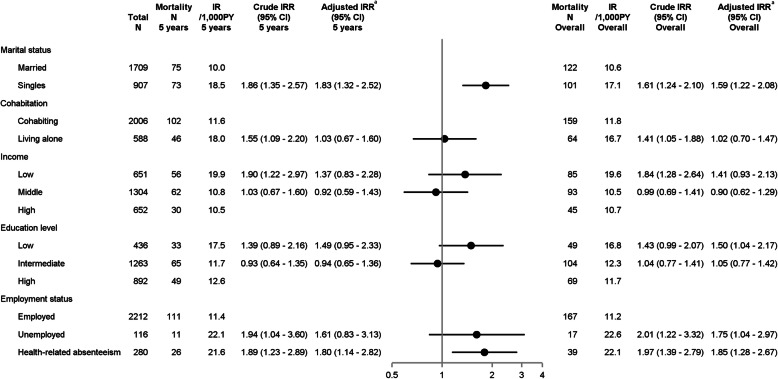
Incidence rates and incidence rate ratios of mortality by socioeconomic position. Plots illustrate 5-year adjusted IRRs and error bars 95% CIs. Superscript lowercase letter “a” indicates the following: marital status was adjusted for age; cohabitation for age and marital status; income for age, comorbidities, marital status, cohabitation, education, and employment; education for age; employment for age, comorbidities, and education. Abbreviations: CI, confidence interval; IR, incidence rate; IRR, incidence rate ratio; N, numbers; PY, person years

Figure 1 illustrates CIP curves of recurrence and mortality according to SEP. Figures 2 and 3 present IRs and IRRs of recurrence and mortality by SEP, at 5 years, and up to 10 years after breast cancer diagnosis. Unless otherwise stated, the IRRs presented below represent 5-year DAG-adjusted estimates.

Compared with married women, single women had poorer prognosis (IRR recurrence 1.45, 95% CI 1.11–1.89 and IRR mortality 1.83, 95% CI 1.32–2.52). Stratification suggested effect measure modification by ER status whereby single women with ER+ tumors had increased risk of recurrence (IRR 1.60, 95% CI 1.17–2.20) and mortality (1.96, 95% CI 1.35–2.84) (Additional file 1: Tables S3–S4). We observed increased crude recurrence and mortality risk in women living alone, but estimates attenuated after adjustment. Although estimates were imprecise, the recurrence risk remained increased in single women regardless of cohabitation status, but was higher in singles living alone than in cohabiting single women. In contrast, the mortality in cohabiting single women was higher than in single women living alone (data not shown).

We observed increased mortality in women with low education (IRR 1.49, 95% CI 0.95–2.33). Women with ER+ tumors and low income had increased risk of recurrence (IRR 1.28, 95% CI 0.82–2.00) and mortality (IRR 1.57, 95% CI 0.91–2.69) (Additional file 1: Table S4).

Compared with employed women, women with health-related absenteeism had slightly elevated recurrence risk (IRR 1.22, 95% CI 0.80–1.84), and the mortality was 2-fold higher in those with ER+ tumors (IRR 1.99, 95% CI 1.20–3.28), which was not seen in ER tumors. Mortality was increased in unemployed women with ER+ tumors, but estimates were imprecise (IRR 2.15, 95% CI 1. 08–4.26).

Pooling recurrences with BCSMs (*n* = 343) increased the IRR in unemployed women (IRR 1.53, 95% CI 0.96–2.45) (Additional file 1: Figure S6). Restricting to BCSM (*n* = 203) marginally increased IRRs in all low SEP groups, with the most pronounced change among unemployed women (IRR 2.17, 95% CI 1.23–3.83) (Additional file 1: Figure S7). Mortality risk remained stable in women with health-related absenteeism and was null in unemployed when we altered employment status assessment (Additional file 1: Figure S8).

## Discussion

Single women had increased recurrence risk and mortality compared with their married counterparts; this was mainly seen in those with ER+ tumors. We observed increased mortality in women who had low education, low income, were unemployed, or had health-related absenteeism, especially among those with ER+ tumors.

As outlined by Green at al [42], SEP consists of several elements, which cover separate aspects of SEP, that overlap in a shared core dimension of SEP. One single measure may not completely cover the underlying SEP, but our similar findings across several SEP measures support an impact of SEP on breast cancer prognosis, especially mortality. Although we used administrative data assuring high validity, some misclassification of SEP was possible, and other categorizations could possibly have shed light on other aspects of SEP. Some of our estimates were imprecise, but across SEP measures, the lower bound of the 95% CI had the same direction, supporting that the core dimension of SEP may be associated with prognosis. A slightly larger sample size would have increased the precision of our estimates.

Our findings were partly modified by ER status, similar to findings reported by others [43]. A previous study set in the ProBeCaRe cohort showed that 22% of women with ER+ tumors prematurely discontinued their tamoxifen therapy [44]. Early discontinuation increased the recurrence risk, and lower adherence was observed in single women [44]. Tamoxifen is sometimes associated with bothersome side effects, which may impact treatment adherence [45]. Likewise, taxanes incite a wide range of side effects leading to treatment disruption and/or dose reduction, limiting treatment effectiveness [46]. Social support from a partner might influence treatment compliance, particularly among women suffering from side effects. This may have impacted our findings of higher recurrence and mortality among single women. We did not have information on chemotherapy adherence. A study in postmenopausal women shows that discontinuation of chemotherapy is most often due to comorbidities or age, and rarely based on patient request [47]. Our cohort of young patients had a low prevalence of comorbidities and a long life expectancy. We therefore expect it to be rare for them to discontinue a time-limited treatment such as chemotherapy. Still, any discontinuation of therapy could differ by SEP and could be more likely among patients with low SEP [44, 48]. This could have influenced our findings.

Similar to our crude estimates, the aforementioned Danish study on breast cancer recurrence reported lower risks in women living alone, also after adjustment for age, education level, comorbidity, calendar period, tumor and lymph node stage, and adjuvant therapy (chemotherapy and/or radiotherapy) [8]. However, their findings may be affected by residual confounding from other dimensions of SEP, which were not incorporated in their study (e.g., income and employment). We note that adjustment for other SEP measures attenuated our estimates. The previous Danish study used a validated algorithm capturing recurrences in Danish registries [49]. However, their adjusted estimates for education were similar to ours, suggesting non-differentially underreporting in DBCG across SEP categories. As such, our relative estimates may not be affected by the underreported recurrences in DBCG.

Our sensitivity analyses confirmed that deaths in this young population were mainly due to breast cancer. Accordingly, the equivalent mortality, but not recurrence risk, especially across levels of education and employment, may indicate underdetection of recurrences in deprived groups. Danish women with low SEP are less likely to be referred to, and to attend, cancer rehabilitation programs [50, 51], including mammography screening [52]. Recurrences may therefore be underdetected in women with low SEP if they opted out of the follow-up programs. These findings may also enhance the understanding of SEP-related disparities in non-tax-funded healthcare settings, by indicating that inequality in cancer survivorship extend beyond obvious factors like insurance coverage.

Several issues should be considered when interpreting our findings. The main strength of this study is the use of routinely collected registry data, with high validity and completeness [24]. The clinical data registered in the DBCG have high validity [41, 53], and the expected direction and association of prognostic characteristics, e.g. stage, were evident (data not shown). Nonetheless, based on a previous study validating DBCG data against medical records [41], our observed incidence of recurrence may be underestimated.

We used registry-based individual-level measures of SEP, with high validity and completeness [24, 30–32]. As such, our classification of marital status had high validity for marriage and registered relationships [24], but does not capture unregistered cohabitation, which is a common way of living in Denmark [54]. We therefore included cohabitation, which, besides marriage and registered partnerships, also considers two persons of different genders at the same address as cohabiting partners if the age difference is less than 15 years, or if they have children. As such, women with a female roommate or partner would be registered as living alone, leading to misclassification. We identified a group of cohabiting singles; this may include women in unregistered partnerships or divorced women living with their old or a new partner. However, the suggested mediation of cohabitation on marital status was imprecise and presumably a chance finding.

Our findings may be prone to unmeasured confounding. Lundqvist et al. [12] found that reproductive and lifestyle factors contributed to the association between SEP and mortality in breast cancer patients. Naturally, reproductive factors in singles may differ to those in married women. Although we incorporated CCI as a proxy for patient health, we had no information on lifestyle factors, e.g., smoking and diet, which may have influenced our observed associations. However, reproductive and behavioral factors in relation to breast cancer are more relevant in postmenopausal women [55]. The CCI does not include mental disorders, which account for 50% of all long-term sick leave in Denmark [56]. Mental disorders before breast cancer are associated with early retirement [57], suggesting exacerbation after diagnosis. This may have contributed to our findings in women with health-related absenteeism.

The average time from recurrence to death varies by breast cancer subtypes, being longer in women with ER+ disease (~ 2–3 years) and shorter in women with ER− (~ 1–2 years) [58]. Thus, we may have underestimated the 5-year recurrence incidence in our sensitivity analysis where we considered BCSMs as recurrences.

Last, docetaxel was the taxane-compound used during the study period, but this has gradually been replaced by paclitaxel due fewer adverse effects [26]. Docetaxel and paclitaxel exert similar structural and microtubule-stabilizing effects [59] and have similar effectiveness [60]. Therefore, our findings are relevant to current clinical practice.

## Conclusions

In summary, premenopausal women who were single at breast cancer diagnosis were more likely to develop recurrence or mortality. Women with low income, low education, unemployment, or health-related absenteeism also had higher mortality. This suggests that detection of recurrent breast cancer may be differential depending on SEP. Our findings were especially evident among women with ER+ tumors, suggesting that poor tamoxifen adherence may have contributed to our findings. Therefore, supportive care to these women may help them derive optimal benefit from cancer treatment. To assist this, an important future perspective is to better understand the mechanisms underlying this inequality.

## Supplementary Information

**Additional file 1: Figure S1.** Covariate assessment. **Figure S2.** Education categorization. **Figure S3.** DAG. **Figure S4.** Flow diagram. **Figure S5.** CIP-curve. **Figure S6.** Sensitivity analysis: Recurrence incl. BCSM. **Figure S7.** Sensitivity analysis: BCSM, **Figure S8.** Sensitivity analysis: Employment. **Table S1.** Employment status. **Table S2.** Charlson Comorbidity Index. **Table S3.** Recurrence by ER status. **Table S4.** Mortality by ER status.

## Data Availability

The data in the present manuscript derive from nationwide, population-based administrative and medical registries, linked anonymously using a personalized identifier. Procedures for accessing the data will be available from the corresponding author.

## Abbreviations

BCSM: Breast cancer-specific mortality
CCI: Charlson comorbidity index
CI: Confidence interval
CIP: Cumulative incidence proportion
DAG: Directed acyclic graph
DBCG: Danish Breast Cancer Group
ER: Estrogen receptor
HER2: Human epidermal growth factor 2
IR: Incidence rate
IRR: Incidence rate ratio
IQR: Interquartile range
PY: Person years
SEP: Socioeconomic position
TNBC: Triple negative breast cancer
TNM: Tumor, node, metastasis
ProBeCaRe: Predictors of Breast Cancer Recurrence
Q: Quartile
