# Correction: Socioeconomic position and prognosis in premenopausal breast cancer: a population-based cohort study in Denmark

**DOI:** 10.1186/s12916-023-02987-4

**Published:** 2023-08-17

**Authors:** Cathrine Fonnesbech Hjorth, Per Damkier, Bent Ejlertsen, Timothy Lash, Henrik Toft Sørensen, Deirdre Cronin-Fenton

**Affiliations:** 1 Department of Clinical Epidemiology, Department of Clinical Medicine, Aarhus University and Aarhus University Hospital, Olof Palmes Allé 43-45, 8200 Aarhus, N Denmark; 2 Department of Clinical Biochemistry and Pharmacology, Odense University Hospital, J.B. Winsløvs Vej 4, 5000 Odense, Denmark; 3 Department of Clinical Research, University of Southern Denmark, Winsløwparken 19, 5000 Odense, Denmark; 4 Danish Breast Cancer Group, Blegdamsvej 9, 2100 Copenhagen, Denmark; 5 Department of Oncology, University of Copenhagen, Blegdamsvej 9, Rigshospitalet, 2100 Copenhagen, Denmark; 6 Department of Epidemiology, Rollins School of Public Health, Emory University, 1518 Clifton Rd, Atlanta, GA 30322 USA

**Correction:** **BMC Med 19, 235 (2021)**

**https://doi.org/10.1186/s12916-021-02108-z**

In our original article [1] the multivariate models outlining the association of cohabitation with breast cancer recurrence and mortality were over-adjusted, as models also included marital status, which is used to define cohabitation. This has now been corrected.

1. In Fig. 2, the 5 years and overall adjusted IRRs and CIs of recurrence were incorrect and stated as 0.89 (0.61—1.29) and 0.85 (0.60—1.19) in the published manuscript, respectively. The corrected estimates are 1.21 (0.90—1.63) and 1.09 (0.83—1.43).
2. In Fig. 3, the 5 years and overall adjusted IRRs and CIs of mortality were incorrectly stated as 1.03 (0.67—1.60) and 1.02 (0.70—1.47), respectively, instead of 1.55 (1.10—2.20) and 1.41 (1.05—1.88).

The correct figures are given ahead.

**Fig. 2:**
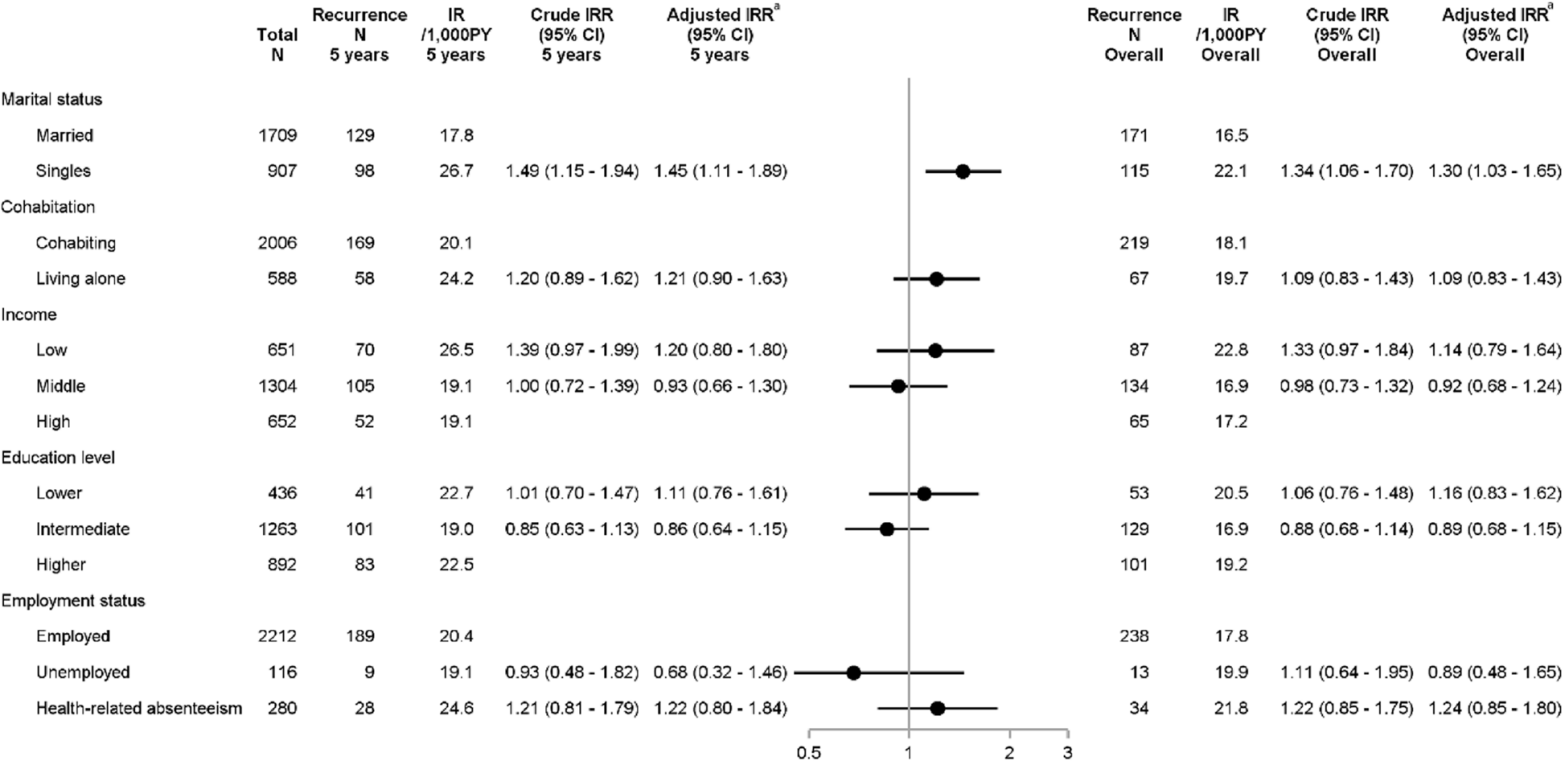
Incidence rates and incidence rate ratios of breast cancer recurrence by socioeconomic position. Plots illustrate 5-year adjusted IRRs and error bars 95% CIs.^a^ Marital status was adjusted for age; cohabitation for age; income for age, comorbidities, marital status, cohabitation, education, and employment; education for age; employment for age, comorbidities, and education. Abbreviations: CI, Confidence Interval; IR, Incidence Rate; IRR, Incidence Rate ratio; N, Numbers; PY, Person-years

**Fig. 3:**
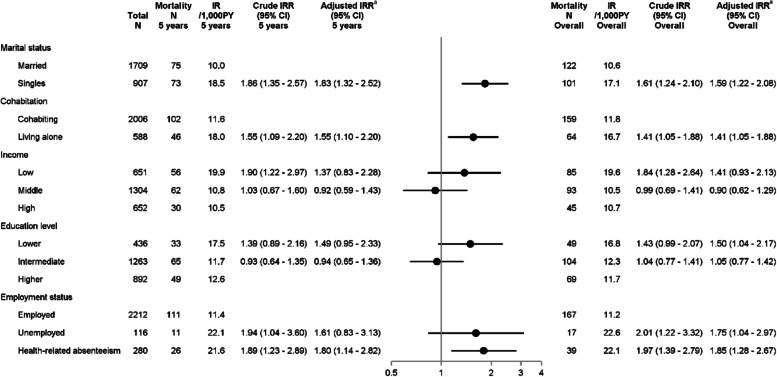
Incidence rates and incidence rate ratios of mortality by socioeconomic position. Plots illustrate 5-year adjusted IRRs and error bars 95% CIs.^a^ Marital status was adjusted for age; cohabitation for age; income for age, comorbidities, marital status, cohabitation, education, and employment; education for age; employment for age, comorbidities, and education. Abbreviations: CI, Confidence Interval; IR, Incidence Rate; IRR, Incidence Rate ratio; N, Numbers; PY, Person-years

We regret the error.
