# Supplementary material for: Socioeconomic position and prognosis in premenopausal breast cancer: a population-based cohort study in Denmark
Source: BMC Med. 2021 Sep 30;19:235. doi: 10.1186/s12916-021-02108-z (PMC8482675; doi:10.1186/s12916-021-02108-z)
Supplement: Supplementary file 1 — Additional file 1: Figure S1. Covariate assessment. Figure S2. Education categorization. Figure S3. DAG. Figure S4. Flow diagram. Figure S5. CIP-curve. Figure S6. Sensitivity analysis: Recurrence incl. BCSM. Figure S7. Sensitivity analysis: BCSM, Figure S8. Sensitivity analysis: Employment. Table S1. Employment status. Table S2. Charlson Comorbidity Index. Table S3. Recurrence by ER status. Table S4. Mortality by ER status. [file 12916_2021_2108_MOESM1_ESM.docx]

**Socioeconomic position and prognosis in premenopausal breast cancer: A population-based cohort study in Denmark**

**SUPPLEMENTAL MATERIAL**

Contents

[Figure S1 - Graphical depiction of underlying time scale and covariate assessment 2](#_Toc79049359)

[Figure S2 - Grouping of education level 2](#_Toc79049360)

[Table S1 - Employment status categorized by weekly entries in DREAM 3](#_Toc79049361)

[Table S2 - Algorithm for Charlson Comorbidity Index 4](#_Toc79049362)

[Figure S3 - Directed acyclic graph (DAG). 5](#_Toc79049363)

[Figure S4 - Flow diagram 6](#_Toc79049364)

[Figure S5 - The cumulative incidence of recurrence and mortality 6](#_Toc79049365)

[Table S3 - Recurrence stratified by ER status 7](#_Toc79049366)

[Table S4 - Mortality stratified by ER status 7](#_Toc79049367)

[Figure S6 - Breast cancer recurrence including breast cancer specific deaths. . 8](#_Toc79049368)

[Figure S7 - Breast cancer specific mortality. . 8](#_Toc79049369)

[Figure S8 - Recurrence and mortality by employment status assessed 1-3 month before breast cancer diagnosis. 9](#_Toc79049370)

# Figure S1 - Graphical depiction of underlying time scale and covariate assessment


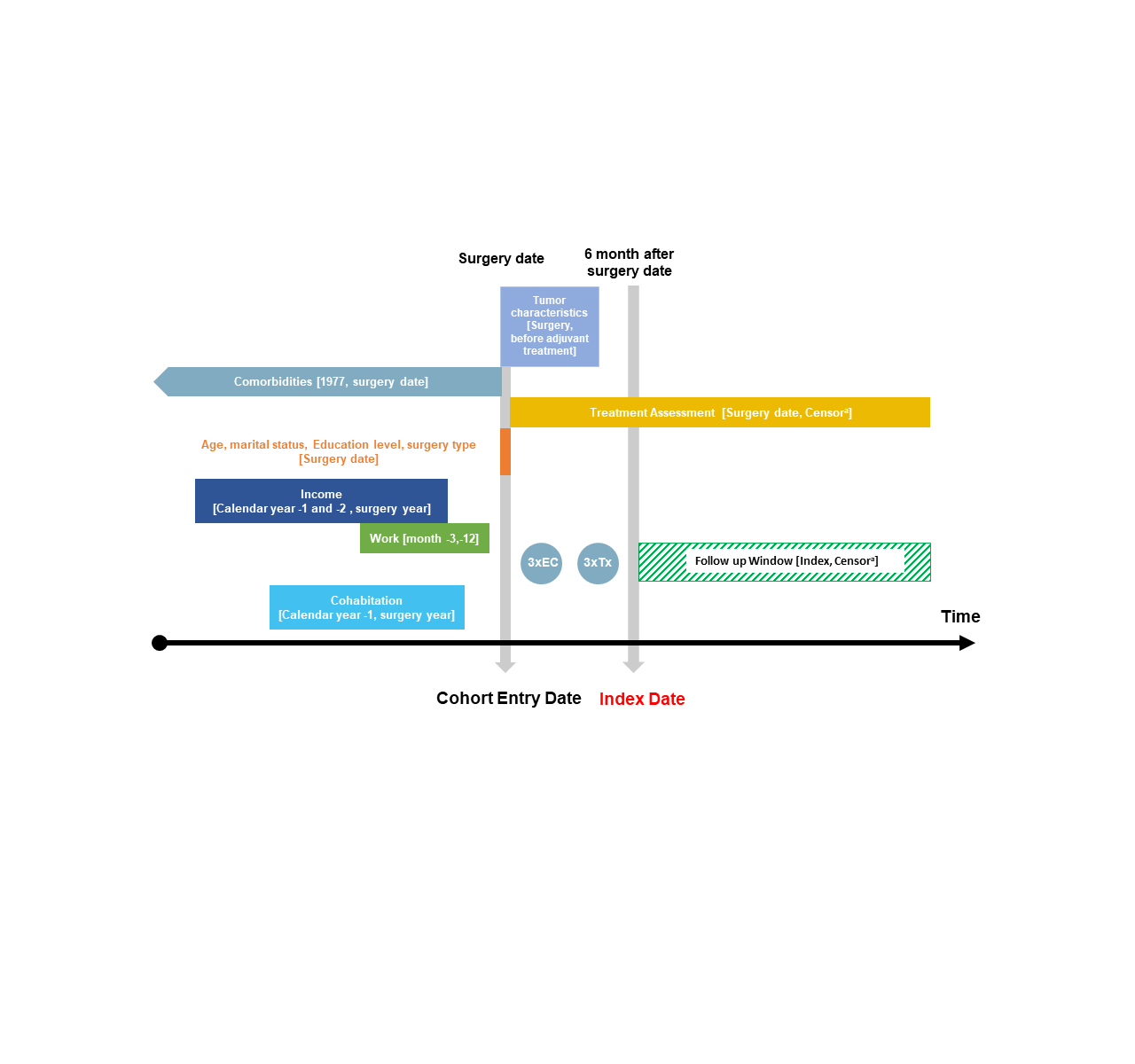


Linkage of individual-patient data from various sources, according to surgery date and index date, including guideline treatment.

Abbreviations: EC = Epirubicin and cyclophosphamide. Tx = Taxane

^a^ In recurrence analysis: earliest of recurrence, death, other malignancy emigration, 10 years or end of study period. In mortality analysis: earliest of death, emigration, 10 years or end of study period

We used a design diagram template by Schneeweiss et al. (35)

# Figure S2 - Grouping of education level according to the International Standard Classification of Education (ISCED 2011) by United Nations Educational, Scientific and Cultural Organization (UNESCO)

| **ISCED level** | **Grouping** | **Level** |
| --- | --- | --- |
| ISCED level 0 – Early childhood education | Elementary school | Low |
| ISCED level 1 – Primary education |  |  |
| ISCED level 2 – Lower secondary education |  |  |
| ISCED level 3 – Upper secondary education | Upper secondary school | Intermediate |
| ISCED level 4 – Post-secondary non-tertiary education |  |  |
| ISCED level 5 – Short-cycle tertiary education |  |  |
| ISCED level 6 – Bachelor’s or equivalent level | Tertiary education | High |
| ISCED level 7 – Master’s or equivalent level |  |  |
| ISCED level 8 – Doctoral or equivalent level |  |  |

Data from the Population’s Education Registry holds data on every person who has attended educational programs authorized by the Danish Ministry of Education, including data on education completed outside Denmark (31).

### Table S1 - Employment status categorized by weekly entries in The Danish Register for Evaluation of Marginalization (DREAM).

No entry reflected that the woman received employer payed salary or was self-supporting. No women in the cohort were retired. Women were categorized with an unknown employment status if not resident in Denmark.

| **Entries** | **Description** | **Employment status** |
| --- | --- | --- |
| No entry | No transfer payment | Employment |
| 521 | Adult trainee |  |
| 651 652 661 662 794 | State Education Fund grants |  |
| 795 | Benefits due to sick child |  |
| 112 113 115 | Unemployment benefit part time |  |
| 122 123 | Vacation payment from employment |  |
| 411-413 | Leave-of-absence schemes |  |
| 121 | Vacation payment |  |
| 881 | Maternity leave pay |  |
| 111 114 | Unemployment benefit all week | Unemployed, not health related |
| 124-126 | Vacation payment from unemployment |  |
| 130-139 141-142 152-153 730-739 741 | Social assistance, not health related |  |
| 160 163-169 | Ready for employment benefit |  |
| 710-719 | Social benefit, immigrants |  |
| 704-709 | Immigration benefit during job training |  |
| 140-149 151 414 700 703 720-729 732 742 751 752 | Education assistance, not health related |  |
| 211-219 221 222 224 225 231 232 297-299 511 522 541 722 759 | Unemployment benefit during special efforts e.g. job training or supervision. |  |
| 761-762 769 771-774 779 782 796 895 | Flexible job (reduced workability) | Health-related work absenteeism |
| 774 890 891-899 | Sick leave benefit |  |
| 740 743-748 | Unemployed awaiting flexible job |  |
| 750 753-758 760 763-768 791 792 | Rehabilitation |  |
| 810 813-819 784 | Vocational rehabilitation program |  |
| 785 870 873-879 | Workability clarification |  |
| 622 781 783 797 | Early retirement pensions |  |
| 611, 621, 793 | Post-employment retirement | Retirement |
| 998 | Retirement |  |
| 997 | Not resident in Denmark | Unknown |

DREAM records weekly information on social security payments to all Danish citizens aged 18 years with high validity and completeness (32).

# Table S2 - Algorithm for Charlson Comorbidity Index containing International Classification of Diseases, 10th revision

|  | **Diseases** | **ICD-10** | **Score** |
| --- | --- | --- | --- |
| 1 | Myocardial infarction | I21;I22;I23 | 1 |
| 2 | Congestive heart failure | I50; I11.0; I13.0; I13.2 | 1 |
| 3 | Peripheral vascular disease | I70; I71; I72; I73; I74; I77 | 1 |
| 4 | Cerebrovascular disease | I60-I69; G45; G46 | 1 |
| 5 | Dementia | F00-F03; F05.1; G30 | 1 |
| 6 | Chronic pulmonary disease | J40-J47; J60-J67; J68.4; J70.1; J70.3; J84.1; J92.0; J96.1; J98.2; J98.3 | 1 |
| 7 | Connective tissue disease | M05; M06; M08; M09; M30; M31; M32; M33; M34; M35; M36; D86 | 1 |
| 8 | Ulcer disease | K22.1; K25-K28 | 1 |
| 9 | Mild liver disease | B18; K70.0-K70.3; K70.9; K71; K73; K74; K76.0 | 1 |
| 10 | Diabetes type1  Diabetes type2 | E10.0, E10.1; E10.9  E11.0; E11.1; E11.9 | 1 |
| 11 | Hemiplegia | G81; G82 | 2 |
| 12 | Moderate to severe renal disease | I12; I13; N00-N05; N07; N11; N14; N17-N19; Q61 | 2 |
| 13 | Diabetes with end-organ damage type1 and type2 | E10.2-E10.8  E11.2-E11.8 | 2 |
| 14 | Any tumor (except BC) | C00-C75 (excluding C50) | 2 |
| 15 | Leukemia | C91-C95 | 2 |
| 16 | Lymphoma | C81-C85; C88; C90; C96 | 2 |
| 17 | Moderate to severe liver disease | B15.0; B16.0; B16.2; B19.0; K70.4; K72; K76.6; I85 | 3 |
| 18 | Metastatic solid tumor | C76-C80 | 6 |
| 19 | AIDS | B21-B24 | 6 |

The Danish National Patient Registry covers all Danish Hospitals and has registered data on all non-psychiatric inpatient admissions and outpatient visits since 1977 (34).

# Figure S3 - Directed acyclic graph (DAG) illustrating confounding and mediation of associations between socioeconomic position (SEP) measures and recurrence and mortality.


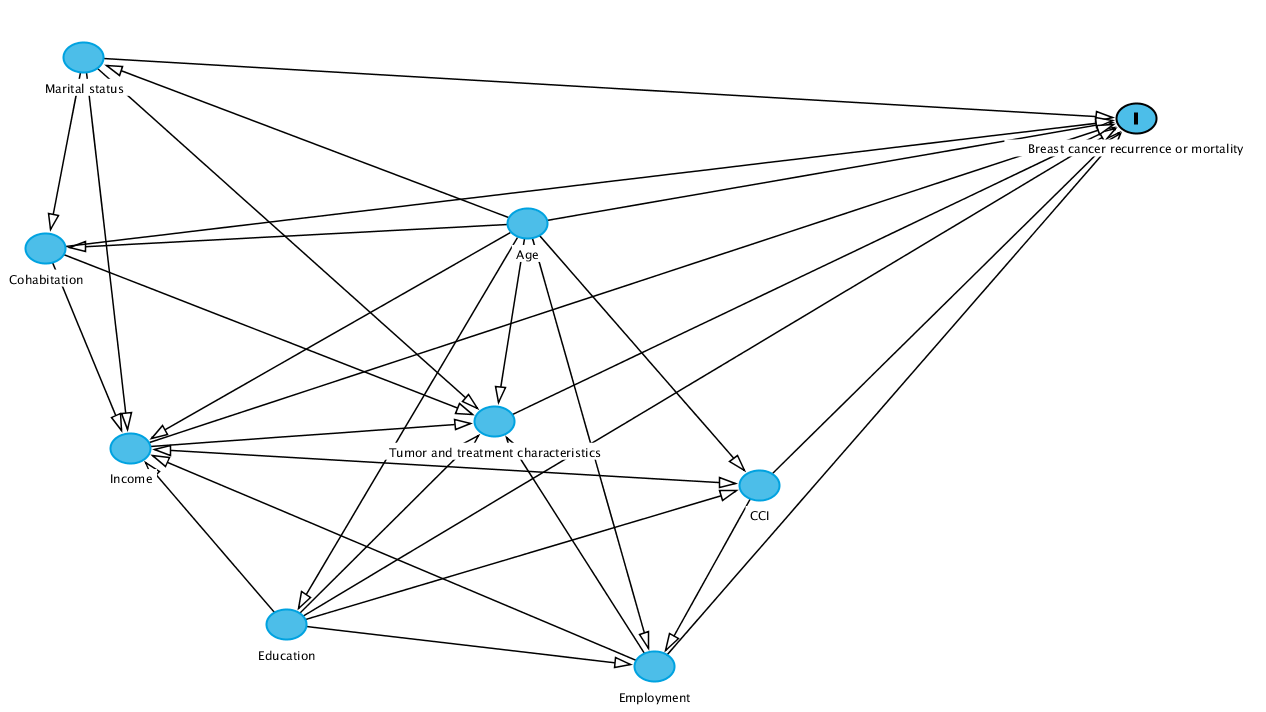


Directed acyclic graph (DAG) based on the anticipated relationships between SEP measures and breast cancer recurrence and mortality, including the other covariates age, CCI and tumor and treatment characteristics (TTC) (aggregated in the figure). For each analysis we considered the given SEP measure as the exposure and breast cancer recurrence or mortality as the outcome. The arrows indicate associations described in the literature of assumed associations based on SEP relationships in Denmark.

Marital status influences: income due to expected higher household income in those married (even though household income is adjusted for number of persons, material purchases are shared with spouse); cohabitation as married women are more likely to live with a partner; TTC due to expected later detection in singles.

Cohabitation influences: income due to expected higher household income in those living with a partner (material purchases require higher payout in women living alone vs. living with a partner); TTC due to expected later detection in women living alone.

Income influences: TTC due to expected lower health seeking behavior/later detection in lower income groups; CCI due to expected poorer lifestyle in lower income groups.

Education influences: income due to expected higher salary by higher education; TTC due to expected higher health seeking behavior in higher educated and; CCI due to expected poorer lifestyle in lower educated; employment due to expected higher rates of unemployed in lower educated.

Employment influences: income due to expected higher household in employed; TTC due to expected lower health seeking behavior in unemployed and women with health related absenteeism.

Age influences: marital status due to expected more singles in younger age groups; cohabitation as younger women are more likely to be living alone; income due to expected low start wages in younger age groups; education as younger women are more likely to be higher educated than older counterparts; employment due to expected higher frequency of unemployment in younger women and more health related absenteeism in older women; CCI as older are more likely to have diagnosed comorbidities; TTC as younger women are more likely to have more aggressive tumors and to receive more aggressive treatments.

CCI influences: income due to expected lower income in women with comorbidities; employment as women with comorbidities are more likely to have health related absenteeism.

Thus, in the Poisson regression models we conditioned marital status on age; cohabitation on age and marital status; income on age, CCI, marital status, cohabitation, employment and education; education on age; employment on age, CCI and education.

DAG created using Dagitty (40)

# Figure S4 - Flow diagram illustrating inclusion and exclusion criteria during study cohort sampling.

| All women in the ProBeCaRe cohort  n=5,959 | | | | |  |
| --- | --- | --- | --- | --- | --- |
|  |  |  | |  |  |
|  |  |  | | Women diagnosed before 2007  n=2980 | |
|  |  |  | |  |  |
|  |  |  | |  |  |
|  | |  | | Women aged >55 years  n=30 | |
|  |  |  |  |  |  |
|  |  |  |  |  |  |
|  |  |  |  | Women not receiving chemotherapy^a^  n=314 | |
|  |  |  |  |  |  |
|  |  |  | |  |  |
|  |  |  | | Women lost to follow-up within 6 months  n=19^b^ | |
|  |  |  | |  |  |
|  |  |  | |  |  |
| Study cohort  Premenopausal women diagnosed with breast cancer 2007-2011 treated with taxane-based chemotherapy  n=2616 | | | | |  |

The study cohort was nested in the ProBeCaRe cohort. During establishment of the ProBeCaRe cohort, ER+ patients not treated with tamoxifen, ER– patients treated with tamoxifen and women with missing information on ER status or tamoxifen were excluded (303 of those were eligible for our study). We excluded women diagnosed before 2007 and women who did not receive chemotherapy. Women listed as premenopausal at primary diagnoses older than 55 years were excluded.

^a^This group was characterized by less severe cancers, compared to the study population, i.e. by higher proportions of stage Ⅰ (61% vs. 26%), grade 1 (34% vs. 15%) and a higher proportion of lumpectomies (74% vs. 61%).

^b^Due to recurrence (n=15), Other malignancy (n=2) or unknown cause (n=2)

# Figure S5 - The cumulative incidence of recurrence and mortality among premenopausal women with non-metastatic breast cancer diagnosis 2007-2011, alive and without recurrence or other malignancy six months after breast cancer diagnosis


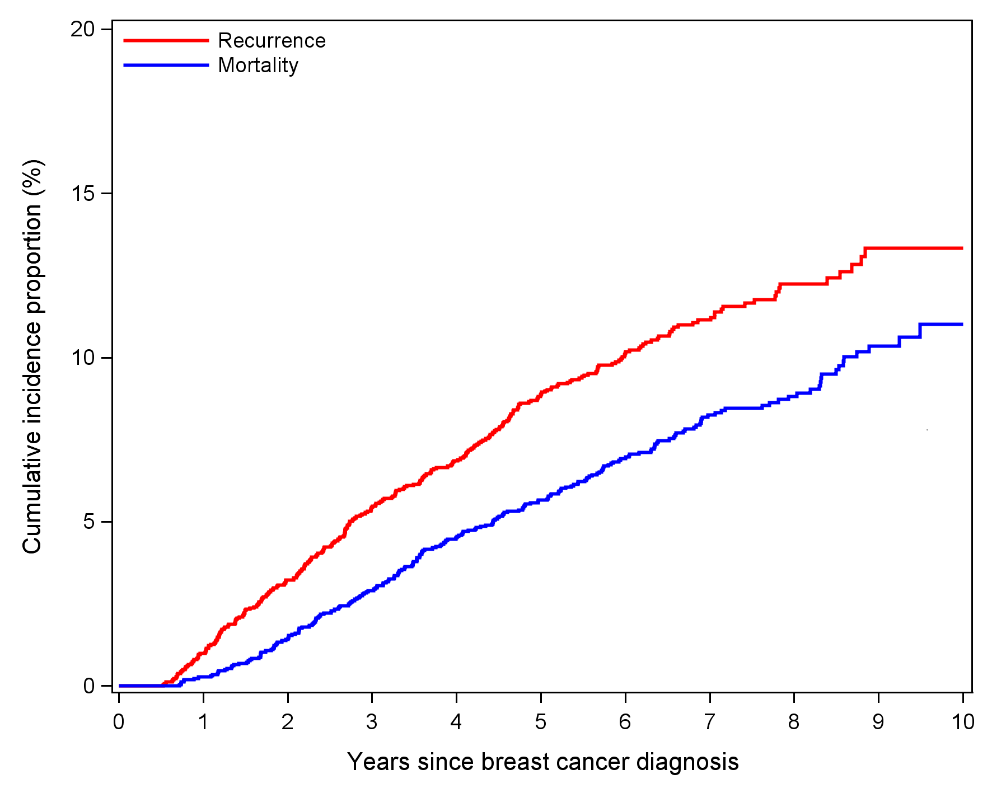


Follow-up started six months after diagnosis, illustrated by the flat curves in this period

# Table S3 - Five year and overall adjusted incidence rate ratio (IRR) of recurrence stratified by ER status

|  | **ER -** | | **ER+** | |
| --- | --- | --- | --- | --- |
|  | **5 years** | **Overall** | **5 years** | **Overall** |
| Marital status |  |  |  |  |
| Married |  |  |  |  |
| Single | 1.14 (0.71 - 1.85) | 1.10 (0.71 - 1.71) | 1.60 (1.17 - 2.20) | 1.36 (1.02 - 1.80) |
| Cohabitation |  |  |  |  |
| Cohabiting |  |  |  |  |
| Living alone | 0.61 (0.30 - 1.25) | 0.69 (0.36 - 1.31) | 1.01 (0.65 - 1.57) | 0.92 (0.61 - 1.37) |
| Income |  |  |  |  |
| Low | 0.75 (0.40 - 1.39) | 0.82 (0.47- 1.45) | 1.28 (0.82 – 2.00) | 1.23 (0.83 - 1.81) |
| Middle | 0.75 (0.42 - 1.35) | 0.89 (0.53 - 1.51) | 1.10 (0.73 - 1.66) | 1.01 (0.70 - 1.45) |
| High |  |  |  |  |
| Education level |  |  |  |  |
| Low | 1.80 (0.92 - 3.54) | 1.60 (0.87 - 2.92) | 0.84 (0.52 - 1.34) | 0.94 (0.62 - 1.41) |
| Intermediate | 1.23 (0.70 - 2.14) | 1.11 (0.68 - 1.82) | 0.72 (0.51 - 1.02) | 0.80 (0.59 - 1.08) |
| High |  |  |  |  |
| Employment status |  |  |  |  |
| Employed |  |  |  |  |
| Unemployed | N/A | N/A | 1.01 (0.46 - 2.21) | 1.27 (0.68- 2.38) |
| Health-related absenteeism | 1.10 (0.54 – 2.59) | 1.38 (0.74 - 2.59) | 1.26 (0.75 – 2.10) | 1.21 (0.77 - 1.92) |

^a^ Marital status was adjusted for age; cohabitation for age and marital status; income for age, CCI, marital status, cohabitation, education and employment; education for age; employment for age, CCI and education based on directed acyclic graphs (see Supplementary Figure 3).

# Table S4 - Five year and overall adjusted IRR of mortality stratified by ER status

|  | **ER -** | | **ER+** | |
| --- | --- | --- | --- | --- |
|  | **5 years** | **Overall** | **5 years** | **Overall** |
| Marital status |  |  |  |  |
| Married | Ref | Ref | Ref | Ref |
| Single | 1.47 (0.97 - 2.24) | 1.40 (0.93 - 2.11) | 1.96 (1.35 - 2.84) | 1.79 (1.27 - 2.54) |
| Cohabitation |  |  |  |  |
| Cohabiting | Ref | Ref | Ref | Ref |
| Living alone | 0.76 (0.42 - 1.37) | 0.84 (0.47 - 1.49) | 0.98 (0.59 - 1.63) | 1.14 (0.70 - 1.84) |
| Income |  |  |  |  |
| Low | 1.00 (0.57 - 1.75) | 0.94 (0.54 - 1.62) | 1.57 (0.91 - 2.69) | 1.49 (0.90 – 2.47) |
| Middle | 0.84 (0.49 - 1.45) | 0.79 (0.46 - 1.36) | 1.25 (0.75 - 2.09) | 1.07 (0.66 - 1.75) |
| High | Ref | Ref | Ref | Ref |
| Education level |  |  |  |  |
| Low | 1.38 (0.78 - 2.44) | 1.33 (0.76 - 2.32) | 1.42 (0.84 - 2.41) | 1.52 (0.92 - 2.50) |
| Intermediate | 0.79 (0.49 - 1.28) | 0.78 (0.49 - 1.25) | 1.07 (0.70 - 1.64) | 1.24 (0.83 - 1.86) |
| High | Ref | Ref | Ref | Ref |
| Employment status |  |  |  |  |
| Employed | Ref | Ref | Ref | Ref |
| Unemployed | 1.23 (0.45 - 3.37) | 1.25 (0.46 - 3.43) | 2.15 (1.08 - 4.26) | 2.33 (1.25 - 4.35) |
| Health-related absenteeism | 0.97 (0.49 - 1.91) | 1.23 (0.65 - 2.32) | 1.99 (1.20 - 3.28) | 2.26 (1.44 - 3.56) |

^a^ Marital status was adjusted for age; cohabitation for age and marital status; income for age, CCI, marital status, cohabitation, education and employment; education for age; employment for age, CCI and education based on directed acyclic graphs (see Supplementary Figure 3).

# Figure S6 - IR and IRR of breast cancer recurrence including breast cancer specific deaths. Plots illustrate 5 year adjusted IRRs and 95% CIs.


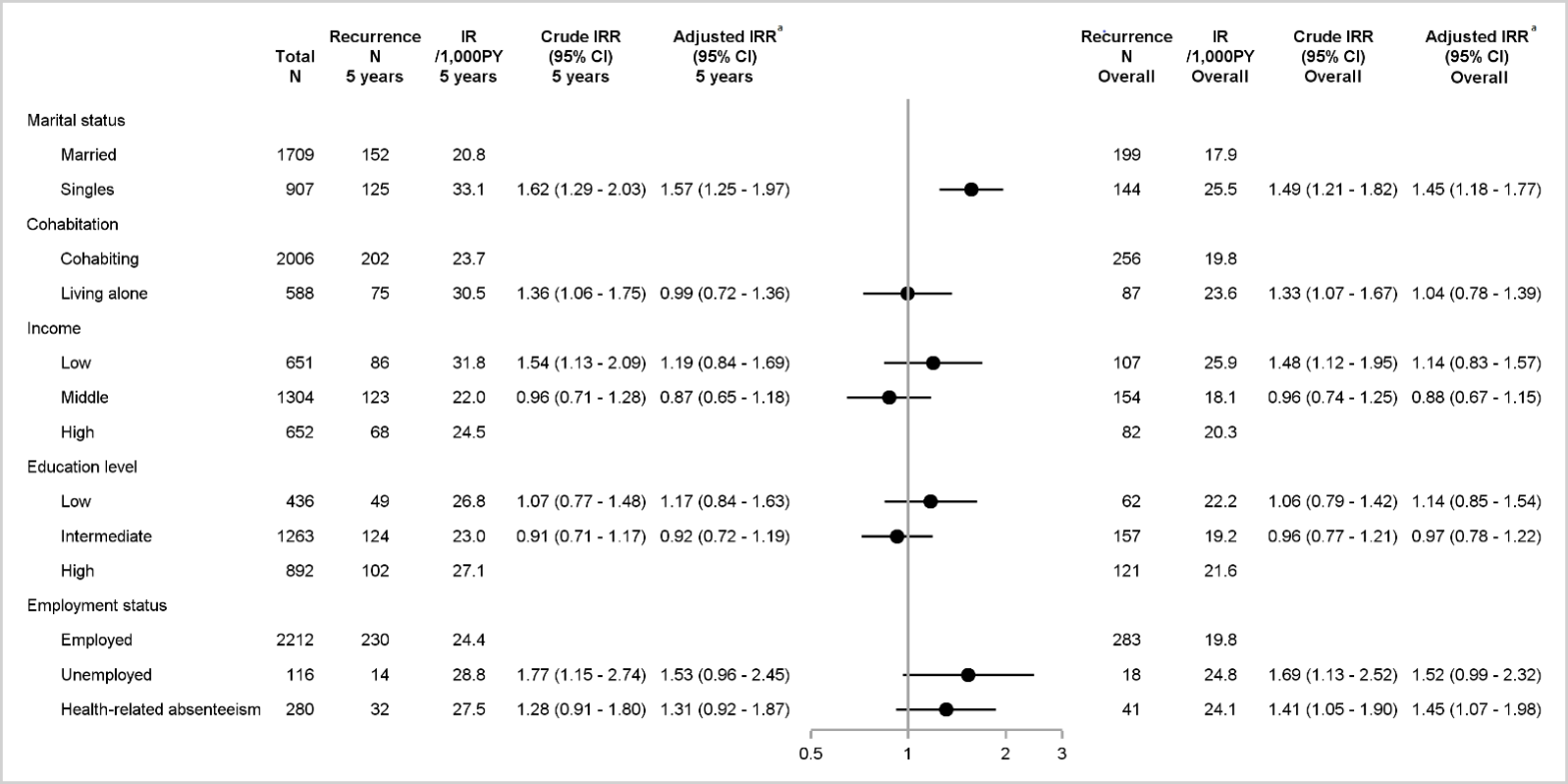


^a^ Marital status was adjusted for age; cohabitation for age and marital status; income for age, CCI, marital status, cohabitation, education and employment; education for age; employment for age, CCI and education based on directed acyclic graphs (see Supplementary Figure 3).

# Figure S7 - IR and IRR of breast cancer specific mortality. Plots illustrate 5 year adjusted IRRs and 95% CIs.

^
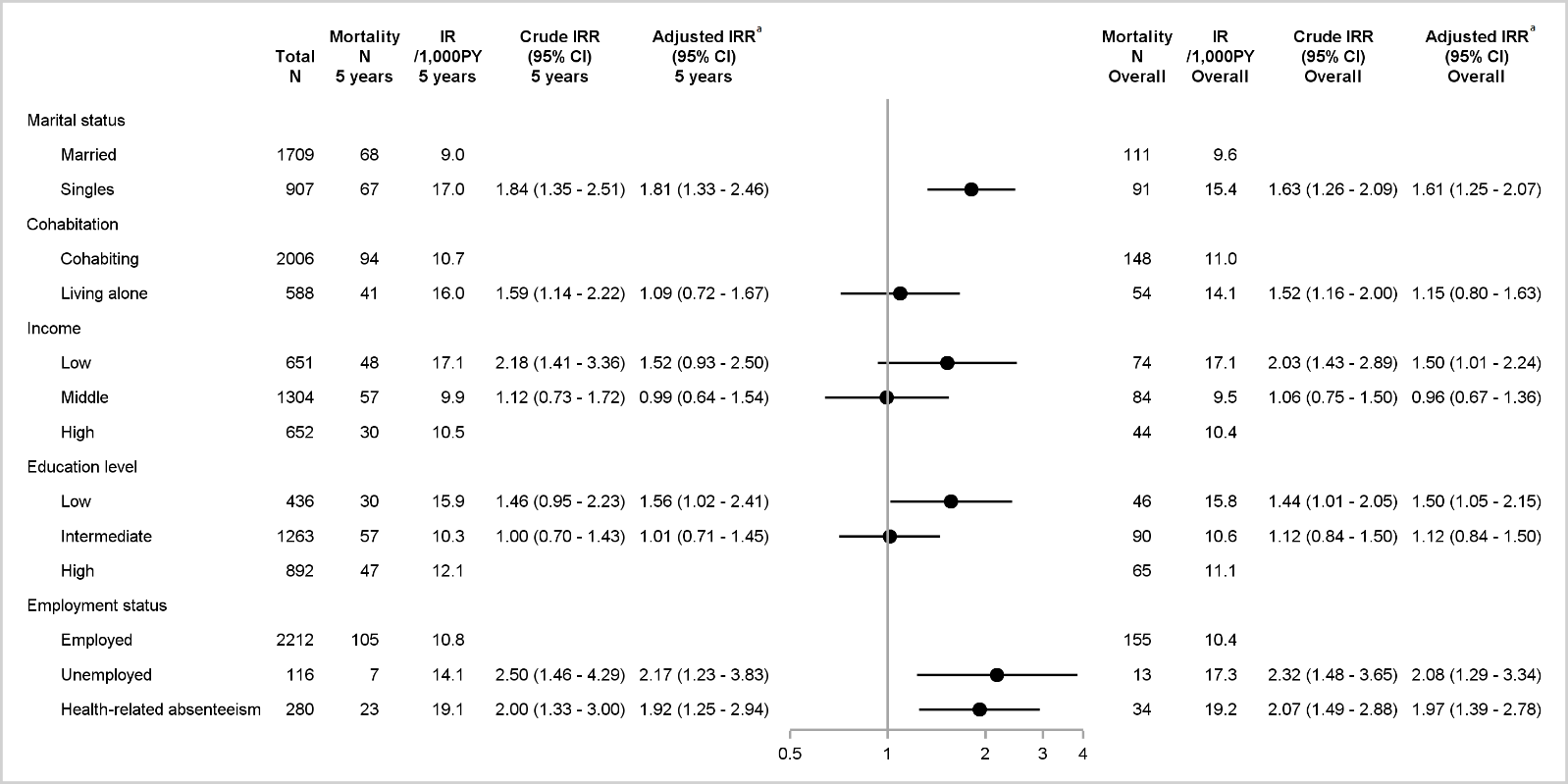
^
^a^ Marital status was adjusted for age; cohabitation for age and marital status; income for age, CCI, marital status, cohabitation, education and employment; education for age; employment for age, CCI and education based on directed acyclic graphs (see Supplementary Figure 3).

# Figure S8 - IR and IRR of breast cancer recurrence and mortality by employment status assessed 1-3 month before breast cancer diagnosis. Plots illustrate 5 year adjusted IRRs and 95% CIs.


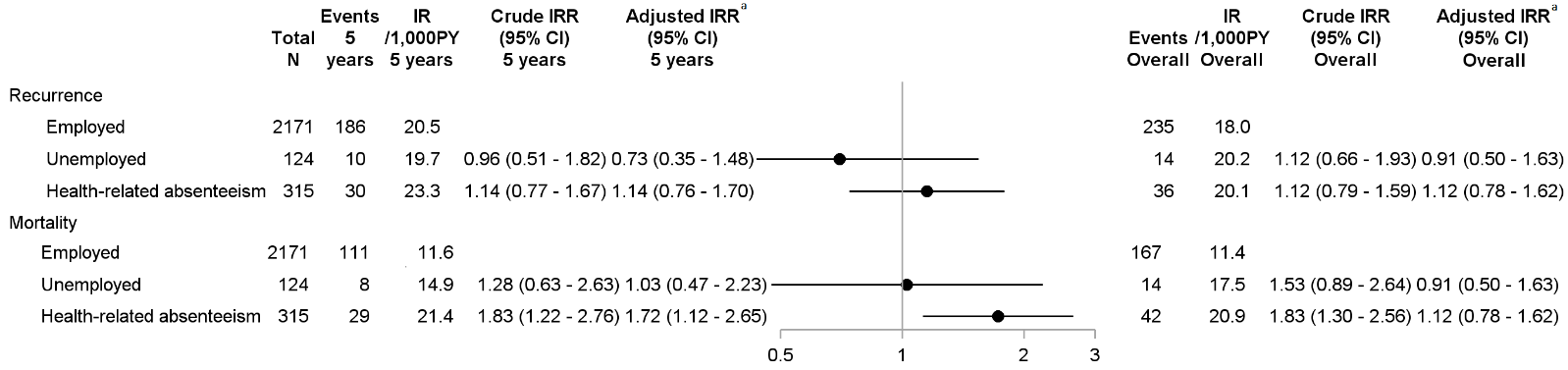


^a^ Adjusted for age, CCI and education based on directed acyclic graphs (see Supplementary Figure 3).
